# Characterization of gut microbiota and metabolites in individuals with constipation-predominant irritable bowel syndrome

**DOI:** 10.3389/fmicb.2025.1617288

**Published:** 2025-09-04

**Authors:** Ya-Li Wang, Xiao-Qian Xu, Yi-Yan Long, Yan-Li Cheng

**Affiliations:** Department of Gastroenterology, The First Hospital of Tsinghua University, Beijing, China

**Keywords:** gut microbiota, irritable bowel syndrome of constipation, metabolomics, metagenomics, dysbiosis

## Abstract

**Objective:**

Constipation-predominant irritable bowel syndrome (IBS-C) is a prevalent functional gastrointestinal disorder with an incompletely understood pathogenesis. Recent studies have found that gut microbiota may contribute to its development. This study aimed to characterize the gut microbiota and associated metabolites in individuals with IBS-C to investigate potential pathogenic mechanisms.

**Methods:**

A total of 22 individuals diagnosed with IBS-C and 22 healthy controls were recruited at the First Hospital of Tsinghua University between January and December 2023. Stool samples were collected and subjected to metagenomic and metabolomic analyses to assess microbial composition and metabolic profiles. Bioinformatic analyses were employed to integrate and interpret the data.

**Results:**

Metagenomic sequencing results indicated no significant differences in overall gut microbial diversity between the IBS-C group and healthy individuals (*p* > 0.05). However, six bacterial species exhibited differential abundance. Notably, the relative abundance of beneficial taxa such as *Megasphaera elsdenii, Bifidobacterium bifidum*, and *Alistipes inops* was significantly reduced in the IBS-C group (*p* < 0.05). Metabolomic profiling demonstrated that differential metabolites were primarily enriched in pathways related to 3 short-chain fatty acids (SCFA) metabolism. SCFA levels were significantly downregulated in individuals with IBS-C, and a trend toward increased levels of the pro-inflammatory metabolite leukotriene D5 was observed.

**Conclusion:**

Individuals with IBS-C demonstrate gut microbiota dysbiosis, characterized by reduced abundance of specific probiotic species and altered SCFA metabolism, along with potential low-grade inflammatory activity. These findings offer insights into the pathophysiological mechanisms of IBS-C and may inform the development of new therapeutic strategies.

## 1 Introduction

Irritable bowel syndrome (IBS) is a prevalent functional gastrointestinal disorder characterized by recurrent abdominal pain accompanied by alterations in bowel movement frequency and/or stool consistency (Liu et al., 2023). IBS is clinically categorized into four subtypes: constipation-predominant (IBS-C), diarrhea-predominant (IBS-D), mixed-type, and unclassified type (Liu et al., 2023). In China, the prevalence of IBS ranges from 1.4% to 11.5%, with IBS-C accounting for approximately one-third of diagnosed cases (Collaboration Group of Functional Gastrointestinal Diseases, Chinese Society of Gastroenterology, Gastrointestinal Motility Group, Chinese Society of Gastroenterology, 2020).

IBS-C is defined by recurrent abdominal pain accompanied by infrequent bowel movements and/or hard stools. These defecation difficulties significantly impact both physical and mental wellbeing, as well as the overall quality of life of affected individuals (Functional Gastroenterology Group, Gastroenterology Society of Chinese Medical Association, Gastrointestinal motility Group, Gastroenterology Society of Chinese Medical Association, 2019). Persistent constipation in individuals with IBS-C increase the risk of complications such as intestinal obstruction, colonic polyps, and tumors of the lower gastrointestinal tract. Moreover, older adults and those with existing cardiovascular disease are at elevated risk of cardiovascular events during episodes of straining associated with defecation (Gallegos-Orozco et al., 2012). These clinical implications underscore the need to elucidate the pathophysiological mechanisms underlying IBS-C and to identify therapeutic interventions.

Advances in gut microbiota research have increasingly implicated microbial dysbiosis as a key factor in the pathogenesis of IBS-C (Liu et al., 2023). The gut microbiota, a major component of the human intestinal microbial ecosystem, exerts extensive influence on host health through the production of metabolites such as short-chain fatty acids (SCFAs), bile acids (BAs), and trimethylamine N-oxide (TMAO) (Zierer et al., 2018). Previous studies have demonstrated that individuals with IBS-D exhibit reduced gut microbiota abundance and diversity, along with decreased production of SCFAs and BAs, thereby promoting disease development (Lo Presti et al., 2019; Undseth et al., 2015; Zhao et al., 2020). However, independent studies specifically targeting microbial and metabolic profiles in IBS-C remain limited. Therefore, a comprehensive characterization of gut microbiota and their metabolites in individuals with IBS-C is warranted and may provide valuable insights for the development of therapeutic strategies.

## 2 Materials and methods

### 2.1 Sample size estimation

The experimental group in this study comprises patients with IBS-C, while the control group consists of healthy individuals with normal bowel movements, matched with the experimental group in terms of age and gender. According to the literature review and the results of the pilot study, the mean fecal calprotectin levels in the control group and the experimental group were 2.805839 and 2.932094, respectively. Since the standard deviation of the mean difference between the groups was unknown, the following formula was used for estimation: $S_{\rho }^{2}=\frac{\left(n1-1\right)S_{1}^{2}+\left(n2-1\right)S_{2}^{2}}{n1+n2-2}$. The estimated standard deviation of the mean difference between the groups was 0.1227818. Subsequently, the power value was calculated using the following function in the R package stats: power.t.test (n, delta, sd, sig.level = 0.05). Ultimately, it was calculated that when the sample size per group was 20, the power to reject the null hypothesis of no difference in fecal calprotectin levels between the groups could reach 90%. Considering a potential dropout and refusal rate of 10%, the minimum number of participants required for both the experimental and control groups was ultimately determined to be 22 each, totaling at least 44 participants to ensure the scientific validity of the study design.

### 2.2 Research participants

This study included 22 individuals diagnosed with IBS-C who presented to the Department of Gastroenterology at the First Hospital of Tsinghua University between January 2023 and January 2024. A control group of 22 healthy individuals with normal bowel habits, matched for age and sex, were recruited during the same period.

Inclusion criteria for the experimental group (IBS-C) were as follows:

(1) Age between 18 and 60 years.
(2) Diagnosis consistent with the Rome IV criteria and China's diagnostic standards for IBS, (Functional Gastroenterology Group, Gastroenterology Society of Chinese Medical Association, Gastrointestinal motility Group, Gastroenterology Society of Chinese Medical Association, 2019) defined as a symptom duration exceeding 6 months, with recurrent abdominal discomfort occurring at least once weekly over the past 3 months, accompanied by at least two of the following: (i) discomfort related to defecation; (ii) changes in stool frequency; and (iii) changes in stool consistency.
(3) Additionally, participants were required to meet at least two of the following criteria during the previous three months (Undseth et al., 2015): (i) average abdominal discomfort score ≥ 1 on the visual analog scale (VAS) (0–10); (ii) defecation frequency ≤ 3 times per week; (iii) straining during ≥ 25% of defecations; (iv) ≥ 25% of stools classified as Bristol type 1 (lumpy stools) or type 2 (hard stools); (v) sensation of incomplete evacuation during ≥ 25% of defecations; (vi) sensation of anorectal obstruction or blockage in ≥ 25% of defecations; and (vii) use of manual maneuvers (such as digital assistance) in ≥ 25% of defecations.

Inclusion criteria for the control group were as follows: Healthy individuals with normal bowel function, and without gastrointestinal complaints.

Exclusion criteria for both groups included: Constipation caused by organic disease and/or medication use; Functional constipation; Other IBS subtypes; Presence of other gastrointestinal diseases; Pregnancy; Cardiopulmonary insufficiency, cerebrovascular disease, mental disorders, or other serious chronic illnesses; Diagnosed metabolic or endocrine disorders affecting gut microbiota (e.g. diabetes mellitus, hyperlipidemia, thyroid disease); use of medications known to affect gut function of or microbiota (e.g. laxatives, prokinetics, probiotics) within the past month; and Current infectious diseases or use of antibiotics within the past month.

The study protocol was reviewed and approved by the Ethics Committee of the First Hospital of Tsinghua University. Written informed consent was obtained from all research participants, and all procedures were conducted in accordance with the ethical provisions outlined in the *Declaration of Helsinki*.

### 2.3 Specimen collection

Following the provision of written informed consent, all participants were instructed to provide stool samples according to standardized collection procedures. Each sample was divided into designated containers, including three 1.5 ml cryovials and one 5 ml cryovial. Samples were stored by research personnel within 2 h of collection. The 1.5 ml cryovials were stored at −80°C, and the 5 ml cryovials were stored at −20°C. All specimens were processed and analyzed in a uniform manner following the completion of sample collection.

### 2.4 Metagenomic testing

(1) DNA extraction: Microbial community genomic DNA and DNA from cultured bacterial isolates were extracted using the DNeasy PowerSoil Kit. DNA concentration and purity were assessed using a NanoDrop2000 UV-Vis spectrophotometer and a Qubit3.0 fluorometer.
(2) Shotgun sequencing: Shotgun metagenomic libraries were prepared using the KAPA HyperPlus Library Preparation Kit, which included DNA fragmentation. Libraries were quantified using KAPA Library Quantification Kit. Libraries were constructed for high-throughput sequencing, yielding paired-end reads of 150 base pairs in both forward and reverse directions. Shotgun sequencing was performed on the Illumina NovaSeq 6000 platform.
(3) Sequencing data quality control: Low-quality reads were filtered from the raw sequencing data. Clean reads were assembled, and gene prediction was carried out using MetaGeneMark. A non-redundant gene catalog was generated using CD-HIT, and clean reads were mapped to the gene catalog using BWA software, with a minimum alignment length threshold of 100 base pairs, to calculate gene abundance. The bacterial species with a *p* < 0.05 for inter-group differences in any of the analyses were selected for subsequent analyses. The mass spectrometer used was a TripleTOF 6600+ system, manufactured by AB Sciex.
(4) Taxonomic analysis: Microbial community composition at various taxonomic levels, including class, order, family, genus, and species was analyzed using Metaphlan2 software.

All raw metagenomic sequencing data were subjected to quality control using the MOCAT2 software. Initially, the raw sequencing reads were processed to remove adapter sequences using Cutadapt software (v1.14, with parameters: -m 30). Subsequently, reads with quality scores below 20 and lengths shorter than 30 bp were filtered out using the SolexaQA package, resulting in clean reads. Finally, the filtered reads were aligned to the host genome using SOAPaligner (v2.21, with parameters: -M 4 -l 30 -v 10) to remove contaminating host reads, yielding high-quality clean data.

### 2.5 Metabolomics testing

1. Metabolite extraction: (1) Stool samples were processed by adding 50% methanol at a ratio of 100 mg to 0.5 ml solvent. (2) Samples were homogenized using a tissue grinder (Brand: QIAGEN Model: TissueLyser LT) at 50 Hz for 1 minute with 3 oscillation cycles. (3) The homogenates were then centrifuged (Centrifuge, Brand: Thermo, Model: Legend Micro 17R) at 13,800g (12,000 rpm) at 4°C for 20 min. A 200 μL aliquot of the resulting supernatant was collected and lyophilized. (4) 150 μL of 50% acetonitrile was added to redissolve the lyophilized residue, followed by a second centrifugation at 13,800 g (12,000 rpm) at 4°C for 20 minutes. (5) The final supernatant was transferred into sample vials for analysis. (6) Quality control samples were prepared in parallel.
2. Instrumental analysis: The chromatography column (Waters HSS T3 column) was equilibrated prior to sample injection. Samples were injected only after the instrument stabilized. The detailed information of the materials used is presented in Supplementary Table 1.

The raw mass spectrometry data were imported into Progenesis QI (version 2.4.691127652) for processing. Within Progenesis QI, the built-in automated workflow was executed, which primarily includes background noise filtering, deconvolution of spectra, retention time drift correction, and peak detection/alignment, as well as normalization of peak response intensity across samples. After processing with Progenesis QI, a data matrix containing the feature list (with retention time and mass-to-charge ratio) and corresponding normalized peak areas/intensity values was exported. The subsequent data processing steps (performed outside Progenesis QI) are as follows:

1) Missing Value Imputation: Missing values in the exported data matrix were imputed using the minimum value imputation method.
2) Data Filtering Based on QC Samples: The reproducibility of the data was assessed using quality control (QC) samples. The coefficient of variation (CV) for each feature was calculated across the QC samples. Differential metabolites were identified using a three-tiered approach: (1) Features with inter-QC CV > 30% were removed; (2) Variable importance in projection (VIP) scores > 1 from Orthogonal partial least-squares discriminant analysis (OPLS-DA) models (SIMCA-P v16.0) were retained; (3) Univariate tests (Welch's t-test/Mann-Whitney U) with FDR-adjusted *p* < 0.05 were applied. Metabolites fulfilling all three criteria were designated as differential, to ensure that subsequent analyses were based on highly reproducible data.

OPLS-DA was employed to filter out signals unrelated to classification, thereby constructing the OPLS-DA model. The quality of the model was assessed using cross-validation, yielding R2Y and Q2 values. R2Y represents the proportion of variance in the response variable that is predictable from the predictors, while Q2 reflects the model's predictive ability. These metrics enable the evaluation of model performance. Through this model analysis, metabolites were scored based on their VIP. Metabolites with higher VIP scores are considered to have greater contributions to group differentiation (Supplementary Figure 1).

### 2.6 Statistical analysis

Statistical analyses were conducted using SPSS version 26.0. For continuous variables that conformed to a normal distribution, data were expressed as mean ± standard deviation (SD), and comparisons between groups were performed using the independent samples *t*-test. For continuous variables that did not meet the assumptions of normality or homogeneity of variance, data were expressed as medians with interquartile ranges, and inter-group comparisons were performed using non-parametric tests. Categorical variables were analyzed using the chi-squared test. Alpha diversity analysis was performed at the species level to assess the within-sample microbial diversity. The Shannon, Simpson, Inverse Simpson, Richness, and Evenness indices were calculated using the diversity function from the vegan package in R. The specific calculation method for β-diversity involved using the pcoa function in R to sort the eigenvalues and eigenvectors, selecting the top eigenvalues to represent the distance differences between samples. The significance of community differences between groups was assessed using PERMANOVA (Permutational Multivariate Analysis of Variance). The PERMANOVA analysis was performed using the adonis2 function from the vegan package, with *P*-values obtained through 1,000 permutations. The differences in alpha diversity intergroup were assessed using the non-parametric Wilcoxon rank-sum test, implemented with the wilcox.test function in R. OPLS-DA was performed to assess group separation and generate VIP scores. Model validity was confirmed via permutation testing (n=200 iterations, R^2^Y and Q^2^ intercepts < 0.05). A *p* < 0.05 was considered to indicate statistical significance.

## 3 Results

### 3.1 Baseline data analysis

A total of 44 participants were enrolled in the study, comprising 22 individuals in the IBS-C group and 22 individuals in the control group. Table 1 provides a summary of the demographic characteristics and the baseline comparisons between the two groups. No statistically significant differences were observed in age or sex distribution between the two groups (*p* > 0.05).

**Table 1:** Comparative analysis of baseline data between the IBS-C and control group.

**Baseline data**	**IBS-C (*n* = 22)**	**Control group (*n* = 22)**	**x**	**t**	***P***
Age (years)	39.00 ± 11.43	37.91 ± 8.78		−0.355	0.724
Sex			0.121		0.728
Male	5 (22.73%)	6 (27.27%)			
Female	17 (77.27%)	16 (72.73%)			

### 3.2 Gut microbiota metagenomic testing results

#### 3.2.1 Comparison of α diversity and β diversity between groups

**Figure 1:**
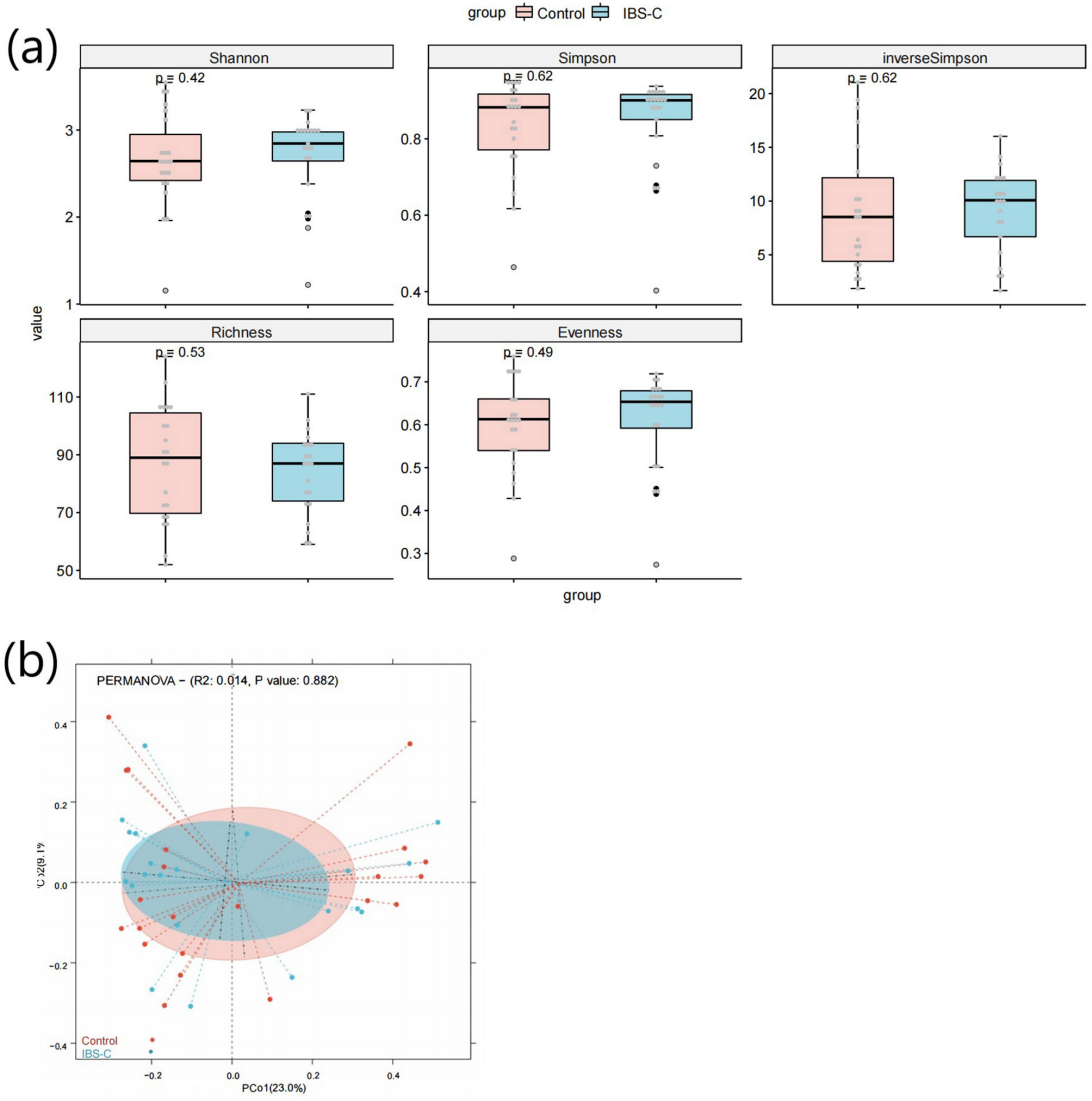
**(a)** Box plot comparing α-diversity indices of gut microbiota between the IBS-C and control groups. (a) shows the box plot of the Shannon index calculation results for species diversity between groups; (b) shows the box plot of the Simpson index calculation results for species diversity between groups; (c) shows the box plot of the inverse Simpson index calculation results for species diversity between groups; (d) shows the box plot of the Richness index calculation results for species diversity between groups; (e) shows the box plot of the Evenness index calculation results for species diversity between groups. The horizontal axis in each figure represents the group names, while the vertical axis represents the diversity values. The three horizontal lines within each box represent the upper quartile, median, and lower quartile, respectively. The two lines extending from the ends of the box indicate the distribution range of normal values for that batch of data, with data points outside this range considered as outliers. The *P*-value in the upper left corner represents the *P*-value obtained from the significance test for differences. **(b)** PCoA plot based on microbial species composition, illustrating β-diversity between groups. The horizontal axis represents the first principal coordinate, while the vertical axis represents the second coordinate. Each point corresponds to an individual sample, with samples of the same color belonging to the same group. The distance between samples within the same group indicates the degree of reproducibility, with closer distances suggesting stronger reproducibility. The distance between samples from different groups reflects the differences between groups, with greater distances indicating higher heterogeneity.

α diversity refers to the microbial diversity within an individual sample, reflecting species richness, evenness, and overall diversity (Xia et al., 2018). Comparative analysis of α-diversity indices, such as the Shannon and Simpson indices between the IBS-C group and control group showed no statistically significant differences (*p* > 0.05, Figure 1a), suggesting comparable microbial diversity within samples across both groups. β diversity evaluates differences in microbial community composition between groups and was assessed using Principal Co-ordinates Analysis (PCoA) (Xia et al., 2018). No statistically significant differences in β diversity were observed between the IBS-C and control groups (*p* > 0.05, Figure 1b).

#### 3.2.2 Analysis of gut microbiota composition between groups

**Figure 2:**
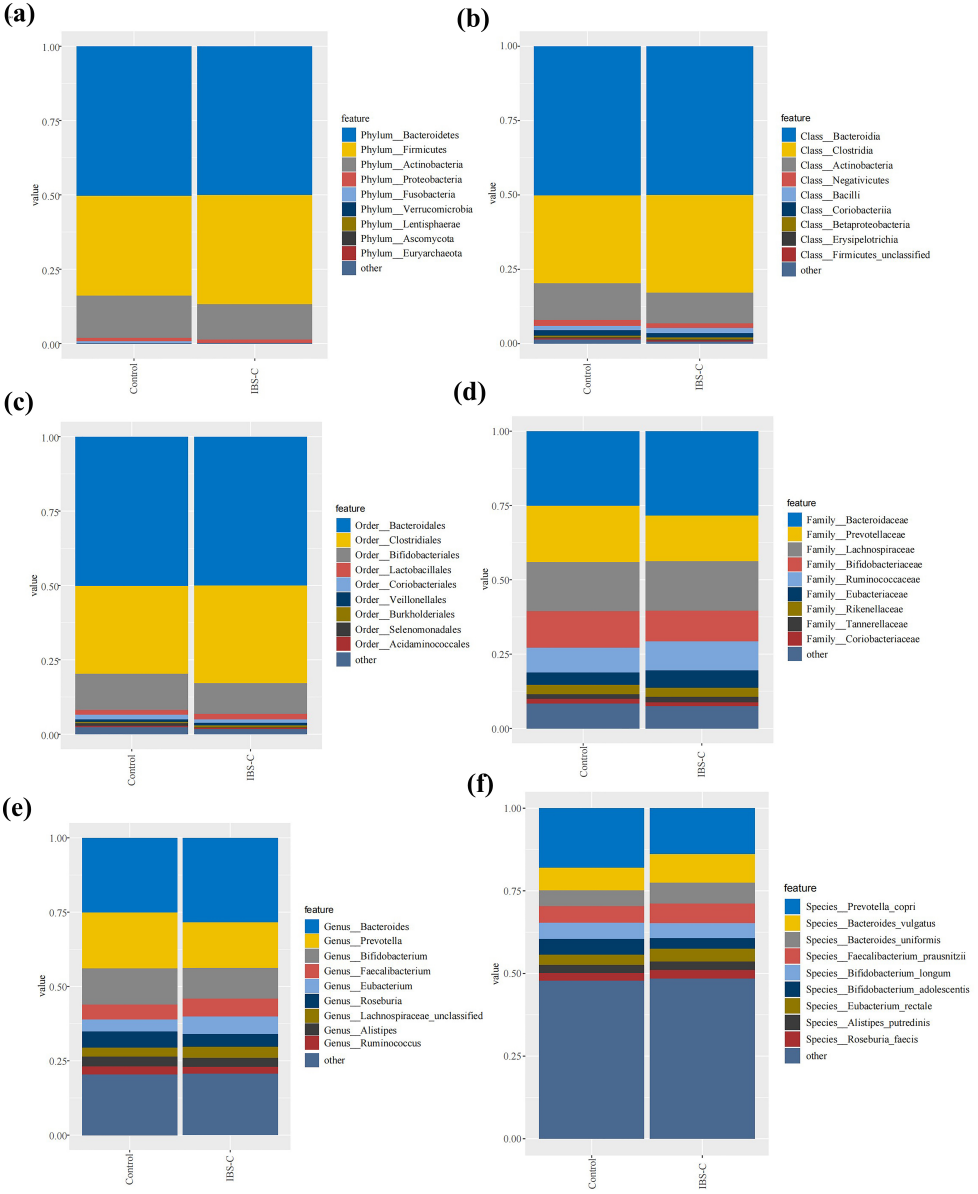
Composition of gut microbiota between the IBS-C and control groups at various taxonomic levels: **(a)** Phylum level; **(b)** Class level; **(c)** Order level; **(d)** Family level; **(e)** Genus level; **(f)** Species level.

Metagenomic data were annotated across multiple levels, including phylum, class, order, family, genus, and species. Analyses were primarily conducted at the phylum, genus, and species levels. At the phylum level, the most abundant bacterial taxa in both groups were Bacteroidetes, Firmicutes, and Actinobacteria in descending order of relative abundance. No statistically significant differences in the relative abundance of these phyla were observed between the two groups (Figure 2).

At the genus level, the composition of bacterial genera were generally similar between the two groups; however, several genera exhibited differential abundance. Notably, genera such as *Bacteroides* and *Faecalibacterium* demonstrated higher relative abundance in the IBS-C group than in the control group, while *Prevotella* and *Bifidobacterium* were more abundant in the control group.

At the species level, more pronounced differences in bacterial abundance were detected. Species such as *Bacteroides vulgatus* and *Bacteroides uniformis* were more abundant in the IBS-C group, whereas *Prevotella copri* and *Bifidobacterium longum* were present at higher levels in the control group. These findings indicate that while differences in gut microbiota composition between groups were minimal at the phylum level, more distinct variations emerged at the genus and species levels, suggesting species-level shifts may play a role in the pathophysiology of IBS-C.

#### 3.2.3 Differential microbiota enrichment analysis between groups

##### 3.2.3.1 Identification of differential bacterial species

A total of 6 bacterial species were identified as differentially abundant between the IBS-C and control groups. These included *Megasphaera elsdenii, Bifidobacterium bifidum, Klebsiella variicola*, an unnamed *Alistipes inops, Klebsiella quasipneumoniae*, and *Lactobacillus iners*.

##### 3.2.3.2 Differential microbiota enrichment analysis between groups

**Figure 3:**
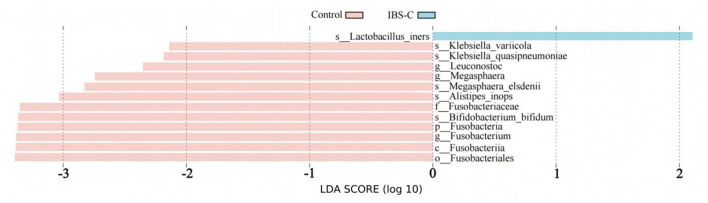
Linear discriminant analysis effect size (LefSe) bar chart showing differentially enriched bacterial species between the IBS-C and control groups. Experimental 1 represents the experimental group, and Control represents the control group. The length of the bars in the bar chart is positively correlated with the magnitude of the impact of the differential species.

Based on Lefse analysis of differential microbiota between groups, differential bacterial species enriched in the control group included: *M. elsdenii, B. bifidum, K. variicola, A. inops*, and *K. quasipneumoniae*. Among them, Bifidobacterium bifidum had the highest LDA (Linear Discriminant Analysis) value, which was 3.36. The differential bacterial species enriched in the IBS-C group was *L. iners*, the LDA value is 2.10 (see Figure 3).

#### 3.2.4 Enrichment analysis of differential microbiota

**Figure 4:**
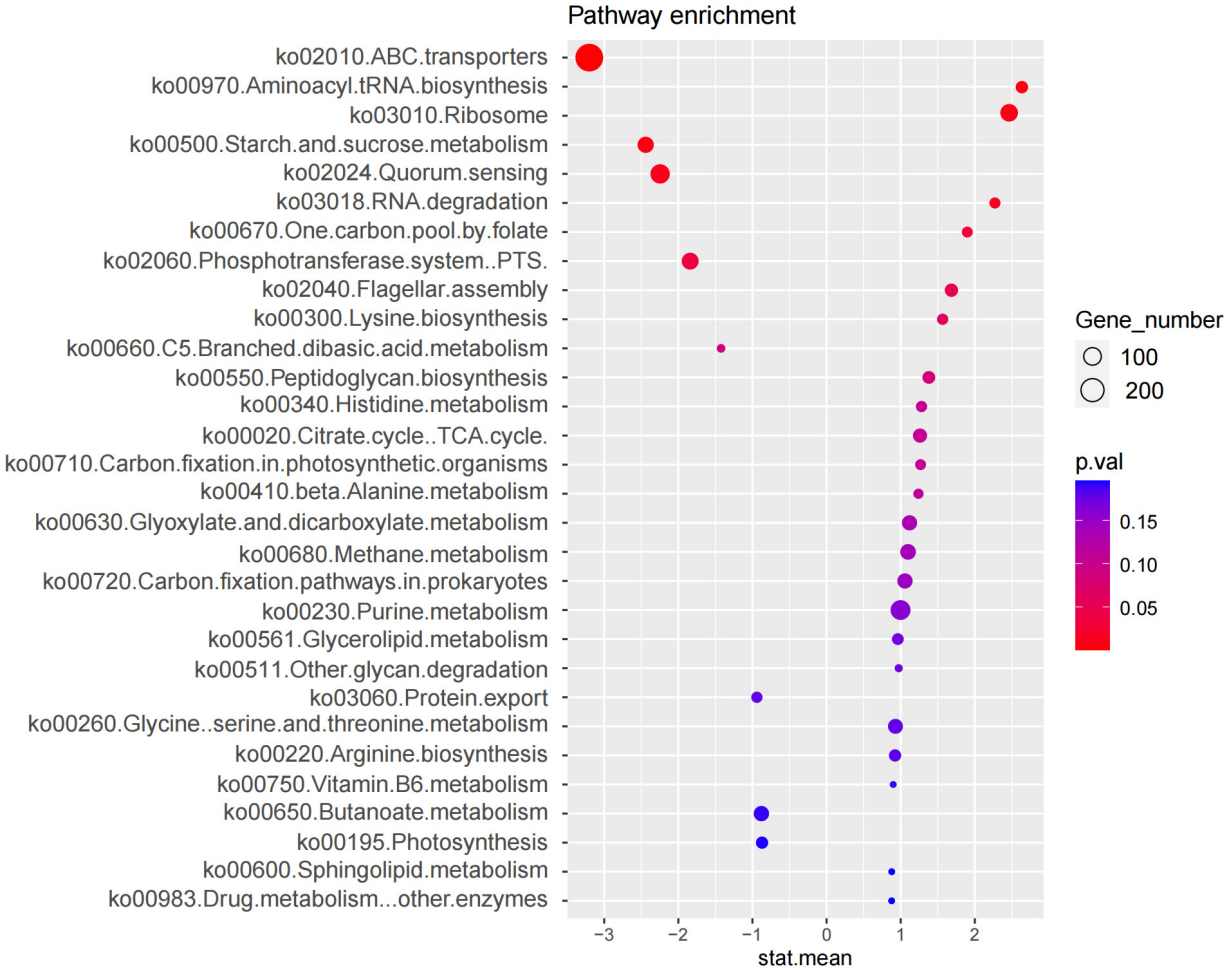
Enrichment analysis of microbial functional pathways based on KEGG annotation. The x-axis (stat.mean) represents the magnitude of differential gene abundance; the y-axis indicates the pathway name. The bubbles represent the number of genes enriched in the corresponding pathways, with larger bubble diameters indicating a greater number of enriched genes. The color of the bubbles represents the *P*-value.

Functional prediction based on the Kyoto Encyclopedia of Genes and Genomes (KEGG) database was performed to analyze pathways enriched by differentially abundant bacterial species (Figure 4). Nine KEGG pathways were found to differ significantly between the two groups (*p* < 0.05) (Table 2), with five pathways upregulated and four downregulated in the IBS-C group compared to the control group. Upregulated pathways in the IBS-C group included: (1) ko00970—Aminoacyl tRNA biosynthesis (Rubio Gomez and Ibba, 2020): Involved in the translation process by facilitating amino acid attachment to tRNA. (2) ko03010—Ribosome (Ben-Shem et al., 2011): Encodes components of the ribosomal machinery, contributing to protein synthesis. (3) ko03018—RNA degradation (Peter et al., 2022): Associated with the turnover of RNA molecules. (4) ko00670—One carbon pool by folate: Participates in cofactor and vitamin metabolism through the folate-mediated transfer of one-carbon units. (5) ko02040—Flagellar assembly (Wen et al., 2023): related to bacterial motility.

**Table 2:** Enrichment analysis of the microbiota differences between the experimental and the control group using KEGG pathways.

**Pathway**	**stat.mean**	***p*.val**	**Experimental**
ko02010.ABC.transporters	−3.201	0.001	Down
ko00970.Aminoacyl.tRNA.biosynthesis	2.635	0.006	Up
ko03010.Ribosome	2.463	0.008	Up
ko00500.Starch.and.sucrose.metabolism	−2.441	0.008	Down
ko02024.Quorum.sensing	−2.246	0.013	Down
ko03018.RNA.degradation	2.272	0.015	Up
ko00670.One.carbon.pool.by.folate	1.898	0.033	Up
ko02060.Phosphotransferase.system..PTS.	−1.8404	0.034	Down
ko02040.Flagellar.assembly	1.684	0.048	Up

The downregulated pathways in the IBS-C group included: (1) ko02010—ABC transporters (Zhu et al., 2023): Involved in ATP-dependent transport of various substrates across cellular membranes. (2) ko00500—Starch and sucrose metabolism (Deng et al., 2022): Associated with the microbial degradation and utilization of complex carbohydrates. (3) ko02024—Quorum sensing (Li et al., 2020): Regulates population-dependent bacterial functions, including virulence, conjugation, and biofilm formation. (4) ko02060—Phosphotransferase system (PTS) (Liang et al., 2024): A primary carbohydrate transport system in bacteria, utilizing phosphoenolpyruvate as a phosphate donor.

### 3.3 Gut microbiota metabolomics testing results

#### 3.3.1 Analysis of differential metabolites between groups

##### 3.3.1.1 Identification of differential metabolites

**Figure 5:**
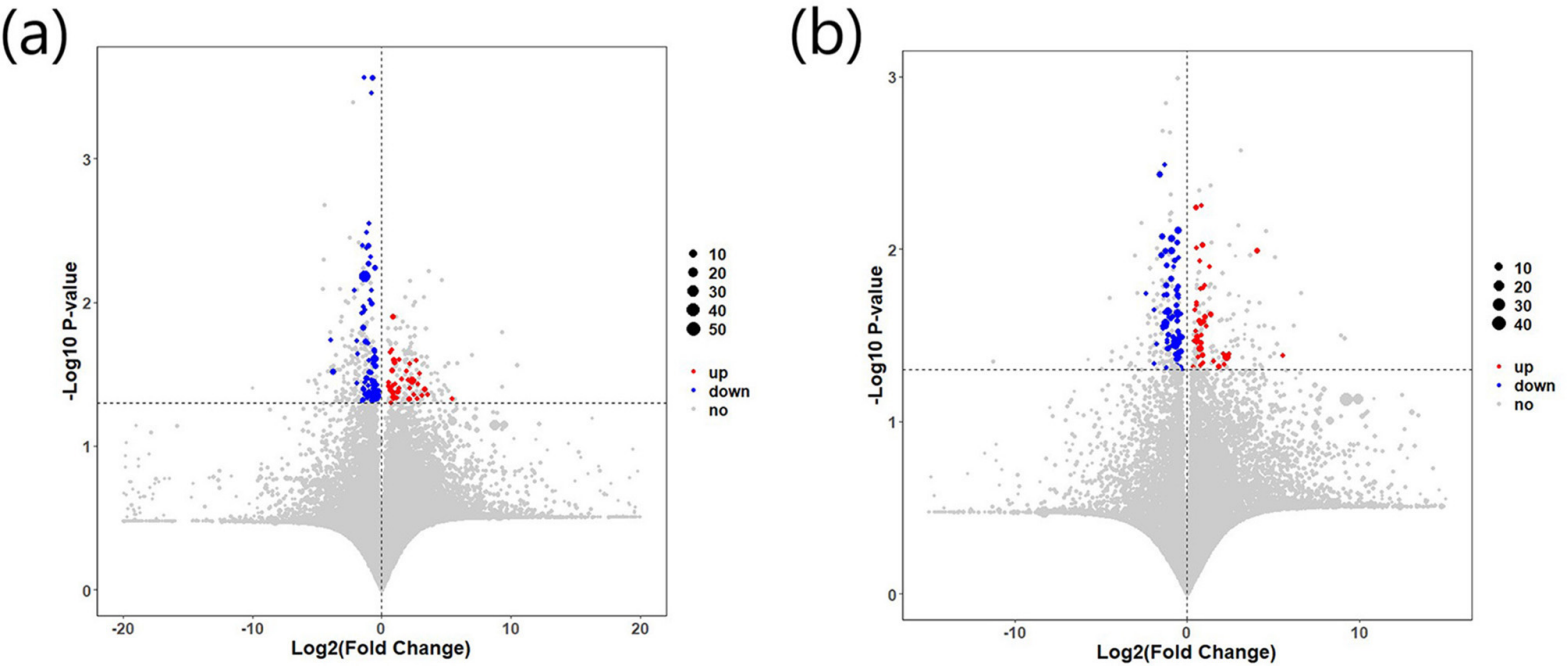
Differential expression of fecal metabolites between the IBS-C and control groups: **(a)** Metabolite expression under negative ion detection mode; **(b)** Metabolic expression under positive ion detection mode. Red indicates upregulated metabolites, while blue indicates downregulated metabolites.

A total of 159 differential metabolites were identified between the IBS-C and control groups. A total of 86 differential metabolites were detected under negative ion detection conditions, including 31 differential metabolites with upregulated expression and 55 differential metabolites with downregulated expression. Under positive ion detection conditions, a total of 73 differential metabolites were identified, including 25 differential metabolites with upregulated expression and 48 differential metabolites with downregulated expression (Figure 5).

##### 3.3.1.2 Characterization of differential metabolites

The identified metabolites were annotated using the Human Metabolome Database (HMDB) and ChemSpider. In the IBS-C group, upregulated metabolites included leukotriene D5 (LTD5), methoxyacetic acid, pyruvic acid, and various intermediates involved in oxidative metabolism. In contrast, downregulated metabolites included N-decanoylglycine, 5-methoxytryptophan, neomycin, deoxycholic acid, propyl valerate glucuronide, and several intermediates associated with SCFA metabolism. Additional metabolites were primarily derived from dietary sources, normal metabolic processes (e.g., lipid and porphyrin metabolism), or were compounds with uncharacterized biological functions that are currently unregistered in major databases.

#### 3.3.2 Analysis of differential metabolic pathways between groups

Given that multiple differential metabolites identified in this study may participate in the same metabolic pathways, pathway analysis was performed using the KEGG database to classify and annotate relevant metabolic routes. A total of 35 metabolic pathways were associated with the differential metabolites. Among these, 32 pathways were classified under the primary category of metabolism, spanning 10 secondary classifications, as follows: nucleotide metabolism (1 pathway), metabolism of terpenoid compounds and polyketide compounds (1 pathway), metabolism of other amino acids (2 pathways), metabolism of cofactors and vitamins (7 pathways), lipid metabolism (1 pathway), global and overview maps (2 pathways), energy metabolism (1 pathway), carbohydrate metabolism (8 pathways), metabolism of other secondary metabolites (1 pathway), and amino acid metabolism (8 pathways). In addition, one metabolic pathway belonged to the environmental information processing category, under the secondary classification of membrane transport. The remaining two pathways were classified under human diseases, specifically related to neurodegenerative diseases (Parkinson's disease) and endocrine and metabolic diseases (type 2 diabetes), respectively (Table 3, Supplementary Figure 2).

**Table 3:** KEGG metabolic pathway classification.

**Class**	**Name**	**Count**	**Annotation**
Human diseases	Neurodegenerative disease	1	Parkinson's disease
	Endocrine and metabolic disease	1	Type II diabetes mellitus
Environmental information processing	Membrane transport	1	ABC transporters
Metabolism	Nucleotide metabolism	1	Pyrimidine metabolism
	Metabolism of terpenoids and polyketides	1	Terpenoid backbone biosynthesis
	Metabolism of other amino acids	2	Taurine and hypotaurine metabolism
			Glutathione metabolism
	Metabolism of cofactors and vitamins	7	Porphyrin and chlorophyll metabolism
			Retinol metabolism
			Pantothenate and CoA biosynthesis
			Vitamin B6 metabolism
			Thiamine metabolism
			Nicotinate and nicotinamide metabolism
			Ubiquinone and other terpenoid-quinone biosynthesis
	Lipid metabolism	1	Steroid biosynthesis
	Global and overview maps	2	Metabolic pathways
			Biosynthesis of secondary metabolites
	Energy metabolism	1	Oxidative phosphorylation
	Carbohydrate metabolism	8	Glycolysis/Gluconeogenesis
			Pyruvate metabolism
			Citrate cycle (TCA cycle)
			Pentose phosphate pathway
			Butanoate metabolism
			Glyoxylate and dicarboxylate metabolism
			Pentose and glucuronate interconversions
			Ascorbate and aldarate metabolism
	Biosynthesis of other secondary metabolites	1	Butirosin and neomycin biosynthesis
	Amino acid metabolism	8	Phenylalanine, tyrosine and tryptophan biosynthesis
			Phenylalanine metabolism
			Arginine and proline metabolism
			Cysteine and methionine metabolism
			Alanine, aspartate and glutamate metabolism
			Valine, leucine and isoleucine biosynthesis
			Glycine, serine and threonine metabolism
			Tyrosine metabolism

**Figure 6:**
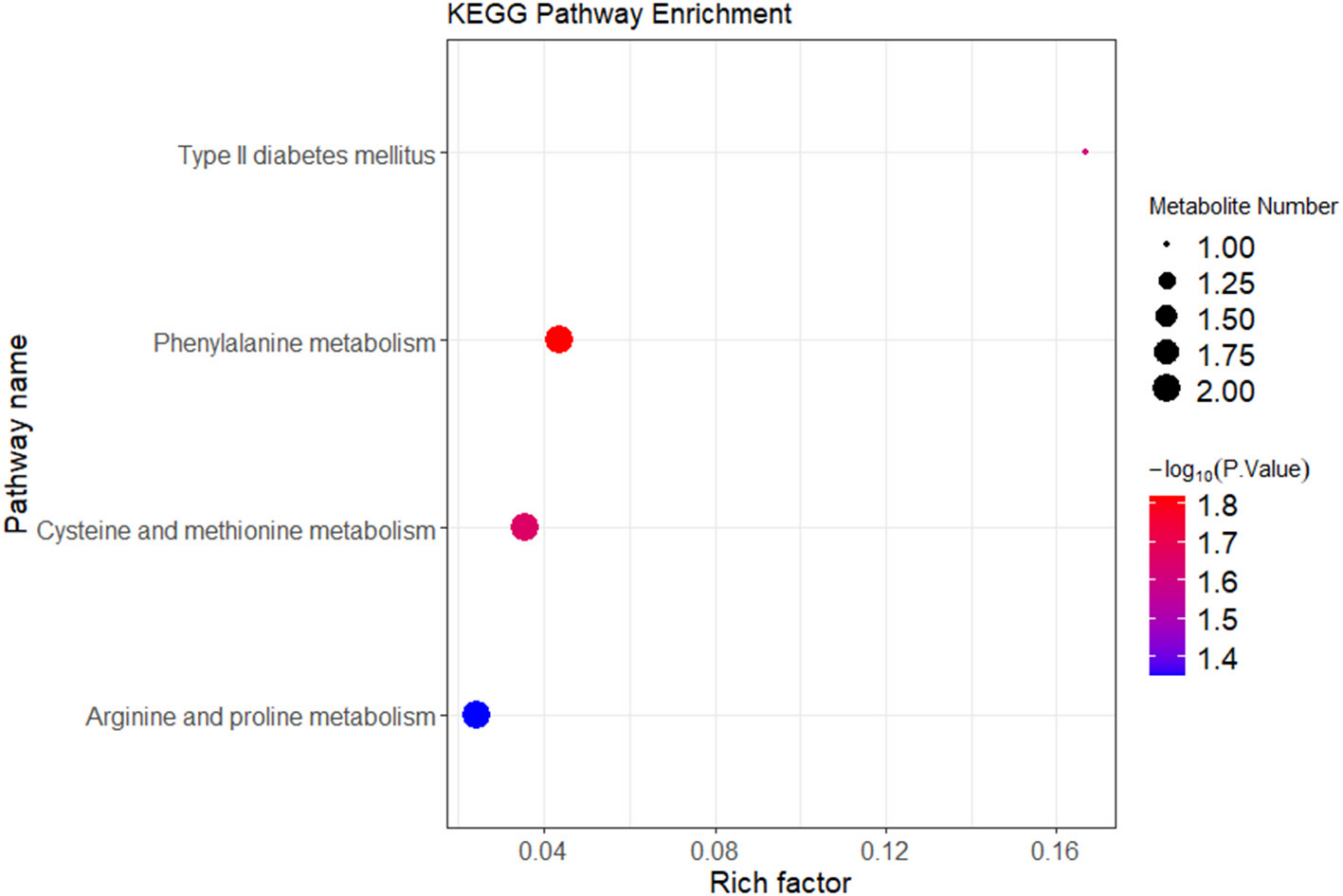
KEGG enrichment analysis of differential metabolites between the IBS-C and control groups. In the figure, each bubble represents a pathway. The horizontal axis indicates the enrichment of differential metabolites within the pathway. The color of the bubble represents the *p*-value from the hypergeometric test. The size of the bubble represents the number of differential metabolites in the corresponding pathway, with larger bubbles indicating a greater number of differential metabolites within the pathway.

Correlation analysis was conducted between the differential metabolites and their associated metabolic pathways. Based on both enrichment scores and the number of associated metabolites, a bubble plot of enriched metabolic pathways was generated (Figure 6). The analysis indicated that differential metabolites were primarily enriched in three key metabolic pathways: Arginine and proline metabolism (involving pyruvic acid and S-adenosylmethioninamine), cysteine and methionine metabolism (also involving S-adenosylmethioninamine), and phenylalanine metabolism (involving pyruvic acid and hydroxyphenylpropionic acid). As illustrated by the metabolic pathway diagram, each metabolic pathway is not isolated. Multiple metabolic pathways intersect at various metabolites, allowing for mutual transformation and influence among them.

#### 3.3.3 Correlation analysis of metagenomic and metabolomic data

**Figure 7:**
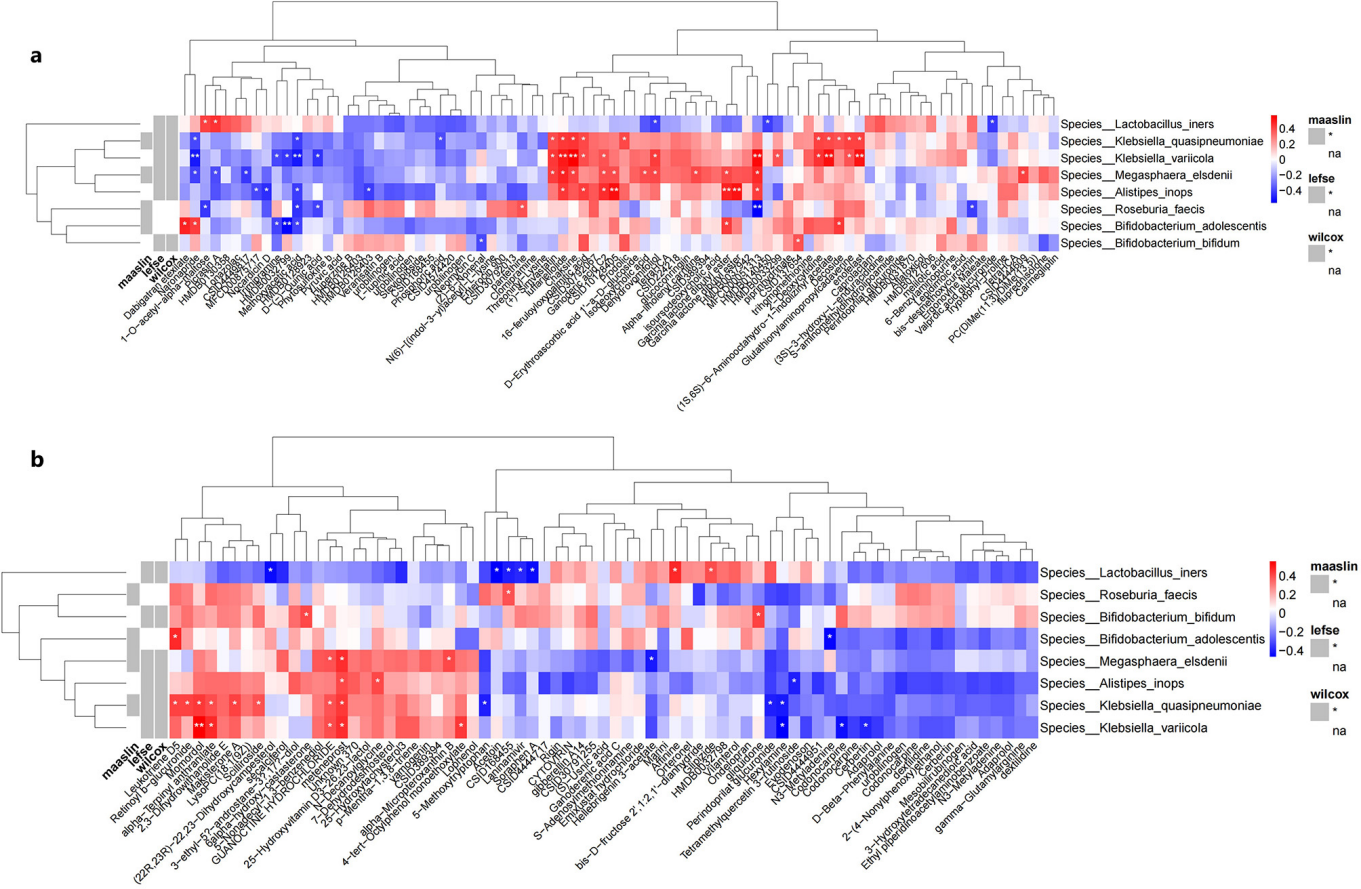
Correlation heatmaps between differential bacterial species and differential metabolites detected under **(a)** negative ion mode and **(b)** positive ion mode. **(a)** shows the heatmap of correlation analysis between differential microbial taxa and metabolites detected under negative ion mode between groups; **(b)** shows the heatmap of correlation analysis between differential microbial taxa and metabolites detected under positive ion mode between groups. Rows represent bacterial species, columns represent metabolites. Red indicates positive correlation, blue indicates negative correlation. *represent the *P*-values from the hypothesis test of the correlation coefficients, with *indicating *P* < 0.05 and **indicating *P* < 0.01.

Spearman's correlation analysis was performed to assess the relationships between the relative abundance of 6 differential bacterial species and the levels of differential metabolites identified in the IBS-C and control groups. A heatmap was generated to visualize these associations. Database annotations of closely related metabolites revealed the following significant correlations: the relative abundance of *M. elsdenii* was positively correlated with the expression levels of acetic acid and isodeoxycholic acid (*p* < 0.05). The relative abundance of *A.inops* was positively correlated with acetic acid levels (*p* < 0.01). The relative abundance of *L. iners* was negatively correlated with the expression of butyric acid (*p* < 0.05) (Figure 7).

## 4 Discussion

This study investigated the gut microbiota profiles of individuals with IBS-C through integrated metagenomic and metabolomic analyses. The findings contribute to a more comprehensive understanding of the microbial and metabolic alterations associated with IBS-C and provide insights into potential mechanisms by which gut microbiota may participate in disease pathogenesis.

At the phylum level, the gut microbiota of both IBS-C and control groups were predominantly composed of *Firmicutes, Bacteroidetes, Actinobacteria*, and *Proteobacteria*, with no significant differences observed in overall species diversity. These results are consistent with previous research findings (Ohkusa et al., 2019). At the species level, six bacterial taxa were found to differ significantly between groups. *M. elsdenii, B. bifidum*, unnamed *A. inops, K. variicola*, and *K. quasipneumoniae* were enriched in the control group, while *L. iners* was enriched in the IBS-C group.

Functional pathway analysis indicated that gene pathways upregulated in the IBS-C group were primarily associated with cell motility and protein translation. This may reflect a microbial gene regulatory response to impaired intestinal motility in IBS-C, leading to upregulated expression of cell motility and proteins needed to relieve constipation-related symptoms (Liu et al., 2022; Rubio Gomez and Ibba, 2020; Aldridge and Hughes, 2002). Gene pathways with downregulated expression in the IBS-C group were involved in carbohydrate uptake and ATP-dependent transport processes. This indicates a reduction in the metabolic capacity of certain gut microbes, particularly those involved in carbohydrate fermentation. which could lead to impaired SCFA production. As SCFAs are essential for maintaining colonic health and regulating motility, this functional deficiency further supports the hypothesis that gut microbiota dysbiosis plays a contributory role in IBS-C pathophysiology (Moore et al., 2023; Thomas and Tampé, 2020; Deutscher et al., 2006).

Our study identified multiple differential metabolites between the two groups, which were enriched in 35 metabolic pathways. Within the carbohydrate metabolism pathways, these metabolites were primarily enriched in pathways related to pyruvate metabolism, butyrate metabolism, glycolysis/gluconeogenesis, the tricarboxylic acid (TCA) cycle, and oxalate and dicarboxylate metabolism, all of which are associated with SCFA metabolism. SCFAs play multiple roles in maintaining gut barrier function, stimulating gut motility, and providing energy for cells, and they are protective factors for IBS-C. Butyrate, an important SCFA, is mainly produced in the gut. It not only promotes gut motility and restores intestinal motility but also serves as the primary energy source for colonic mucosal cells (Mann et al., 2024). In our differential metabolite analysis, we found that acetate and butyrate were downregulated in patients with IBS-C, consistent with previous studies (Mars et al., 2020).

In the amino acid metabolism pathways, we identified three metabolic pathways with significant differences in enrichment: phenylalanine metabolism, arginine and proline metabolism, and cysteine and methionine metabolism. Among these, the phenylalanine metabolism pathway had the highest and most significant enrichment. Although there have been no previous reports on this pathway in IBS-C, studies have shown that *in vitro* experiments with IBS-D, gut microbiota can mediate the catabolism of dietary phenylalanine to produce phenethylamine, which activates the Trace Amine-Associated Receptor 1 (TAAR1) pathway to stimulate the synthesis of 5-HT4 in enterochromaffin cells, thereby promoting gastrointestinal motility and increasing colonic secretion (Zhai et al., 2023). In our study, phenylalanine was downregulated in patients with IBS-C, which may weaken gastrointestinal motility and reduce colonic secretion, leading to the occurrence of IBS-C. This finding may provide a new research direction for the disease.

Additionally, we found that the differential metabolite pyruvate was enriched in multiple metabolic pathways. As a key intermediate in glycolysis, pyruvate can be converted into various compounds such as acetate, ethanol, and butyrate through different enzymatic reactions. Under anaerobic conditions in the gut, certain butyrate-producing bacteria can convert pyruvate to butyrate (Li et al., 2023). In our study, pyruvate was upregulated in the IBS-C group, while butyrate was downregulated. This may be related to the decreased abundance of butyrate-producing bacteria and the reduction of SCFA metabolism-related microbial pathways (e.g., the pathway for pyruvate fermentation to butyrate), ultimately leading to reduced SCFA production and promoting the development of IBS-C.

Correlation analysis between differential bacterial species and differential metabolites indicated that the relative abundance of *M. elsdenii* was positively associated with the expression levels of acetic acid and isodeoxycholic acid. Similarly, abundance of *A. inops* was positively correlated with the expression of acetic acid levels, whereas L. *iners* was negatively correlated with the expression of butyric acid. The correlation analysis between these differential bacterial species and differential metabolites may elucidate their roles in the pathogenesis and progression of IBS-C through pathways such as fatty acid metabolism and amino acid metabolism.

In this study, *M. elsdenii* was identified as a potentially beneficial species associated with SCFA production. Its abundance was positively correlated with the levels of acetic acid, and its relative abundance was significantly reduced in individuals with IBS-C, suggesting that decreased *M. elsdenii* may contribute to diminished SCFA production and impaired gut function. Additionally, *M. elsdenii* abundance was positively correlated with isodeoxycholic acid levels. This finding raises the possibility that *M. elsdenii* may influence bile acid metabolism indirectly, potentially by promoting the growth or activity of bile acid-producing microbiota, thereby influencing the pathogenesis of IBS-C.

Based on the results, a significant reduction in the abundance of *B. bifidum* in individuals with IBS-C was noted in this study. *B. bifidum* has been shown to contain a variety of enzymes involved in mucin degradation, enabling the breakdown of mucin into metabolites such as acetic acid and lactic acid. These metabolites support the growth of beneficial anaerobic gut microbiota and contribute to the improvement of gut microbiota composition and balance (Turroni et al., 2019; Fuyuki et al., 2021). Moreover, mucin degradation by *B. bifidum* does not compromise the intestinal epithelial barrier or promote bacterial translocation. On the contrary, it stimulates mucin synthesis and increases the thickness of the intestinal mucus layer, thereby strengthening the barrier function of the intestinal epithelium (Fuyuki et al., 2021). Additionally, *B. bifidum* has been reported to modulate the toll-like receptor pathway, enhance intestinal epithelial tight junctions, and protect the intestine from inflammation (Nian et al., 2023). Based on these findings, it is proposed that the reduced abundance of *B. bifidum* in IBS-C may lead to decreased SCFA production and impaired intestinal barrier function.

The abundance of *A. inops* was positively correlated with the expression level of acetic acid, and both were reduced in individuals with IBS-C. Previous research has shown that *A. inops* contributes to the maintenance of normal defecation through the production of SCFAs via microbial metabolism. A decline in *A. inops* may therefore result in reduced SCFA production, which could play a role in the onset and progression of IBS-C (El-Salhy, 2023). In addition, based on functional studies of other species within the *Alistipes* genus, it is speculated that *A. inops* may possess tryptophan-hydrolyzing activity (El-Salhy, 2023). Tryptophan is an important precursor in the synthesis of 5-hydroxytryptamine (5-HT), which regulates intestinal motility through the brain-gut-microbiota axis. A reduction in 5-HT synthesis may reduce its regulatory effect on gastrointestinal motility, thereby causing constipation (Wang et al., 2023). Furthermore, this study identified *L. iners* as a differential bacterial species enriched in the IBS-C group. *L. iners* abundance was negatively correlated with butyric acid levels, and individuals with IBS-C exhibited significantly lower butyric acid levels compared to the control group. As a key SCFA, butyric acid deficiency may promote the onset and progression of IBS-C (Rivière et al., 2016).

Furthermore, several significantly altered metabolites were identified in the IBS-C group, although no directly associated differential bacterial species were detected. These metabolites may play a role in the occurrence and development of IBS-C. Upregulated metabolites in the IBS-C group included LTD5, methoxyacetic acid, pyruvic acid, and various intermediate metabolites involved in oxidative metabolism. LTD5 is a member of the D-series LTs, which are classified as eicosanoids (Peters-Golden and Henderson, 2007). Free arachidonic acid (ARA), derived from intestinal epithelial cells and immune cells via phospholipase A2 activity, undergoes oxidation through three enzymatic pathways—cyclooxygenase, lipoxygenase, and cytochrome P450—resulting in the production of eicosanoids including LTs, PGs, and thromboxanes (Peters-Golden and Henderson, 2007; Huang et al., 2021; Moreno, 2017; Goetzl, 2024). LTs have been shown to modulate both intestinal barrier function and immune function. LTD4 and LTB4 promote intestinal epithelial cell survival through Bcl-2 expression (Huang et al., 2021). LTB4 enhances dendritic cell proliferation, migration of chemotaxis mast cells, neutrophil phagocytosis, and kill *Klebsiella* (Huang et al., 2021; Moreno, 2017; Goetzl, 2024; Serezani et al., 2005). However, LTD4 also interacts with cysteinyl leukotriene receptors, thereby increasing intestinal permeability and causing intestinal barrier destruction (Rodríguez-Lagunas et al., 2013). Given the structural and functional similarity between LTD4 and LTD5, the observed upregulation of LTD5 in patients with IBS-C may indicate a similar pro-inflammatory effect, potentially contributing to intestinal inflammation and barrier impairment, consistent with previous findings (Rodríguez-Lagunas et al., 2013; Iancu et al., 2023; Debnath et al., 2021).

In addition to SFCAs, several metabolites were downregulated in the IBS-C group, including Lysophosphatidylcholines (LPCs), propyl valerate glucuronide, and 5-nonadecyl-1,3-benzenediol. LPCs are hemolytic phospholipids that are downregulated in inflammatory conditions such as rheumatoid arthritis, suggesting that low LPC levels are associated with immune dysregulation and inflammation (Wishart et al., 2022). In the context of IBS-C, downregulated LPCs may indicate a state of chronic low-grade intestinal inflammation and immune dysregulation. LPCs also bind to G protein-coupled receptors to induce intracellular calcium mobilization, a process critical for the contractile motility of intestinal epithelial cells (Wishart et al., 2022). Reduced LPC levels may therefore impair calcium signaling, lower calcium ion concentration, leading to decreased intestinal motility, which may further contribute to constipation symptoms in IBS-C. Propyl valerate glucuronide, a glucuronidation metabolite of valproic acid, was also significantly decreased in patients with IBS-C. Valproic acid is commonly used in clinical settings for its anticonvulsant and mood stabilization properties (Gerstner et al., 2008; Wong et al., 2007). The downregulation of propyl valerate glucuronide may reflect altered brain-gut axis signaling and reduced mood-regulating metabolite activity, indirectly supporting the hypothesis that disruption of brain–gut interactions is involved in the pathophysiology of IBS-C.

However, this study has several limitations that warrant consideration. First, although the sample size was statistically powered (90% power) to detect differences in fecal calprotectin levels, the inclusion of only 22 IBS-C patients and 22 controls may limit the generalizability of our findings. Larger cohorts are needed to validate the species-level microbiota alterations (e.g., Megasphaera elsdenii, Alistipes inops) and subtle metabolic shifts observed, particularly given the heterogeneous pathophysiology of IBS-C subtypes (e.g., slow-transit vs. dyssynergic defecation). Second, while we controlled for medications and recent antibiotic use, dietary patterns and lifestyle factors (e.g., fiber intake, physical activity, stress levels) were not quantitatively assessed. These variables significantly influence gut microbiota composition and SCFA production. For instance, low fermentable oligo-, di-, monosaccharide and polyol (FODMAP) diets—commonly adopted by IBS patients—can reduce Bifidobacterium abundance and butyrate synthesis, potentially confounding our results. Future studies should incorporate standardized dietary records and longitudinal sampling to disentangle microbiota-diet-host interactions in IBS-C pathogenesis.

## 5 Conclusion

This study employed an integrated metagenomic and metabolomic multi-omics approach to elucidate the gut microbial and metabolic characteristics of individuals with IBS-C. The findings demonstrate that IBS-C is associated with gut microbiota dysbiosis, which may contribute to disease development through impaired SCFA metabolism and the induction of low-grade intestinal inflammation. The results provide a theoretical foundation for the development of novel therapeutic strategies.

## Data Availability

The original contributions presented in the study are publicly available. This data can be found here: https://www.ncbi.nlm.nih.gov/, accession number PRJNA1306780.
