# Supplementary material for: Characterization of gut microbiota and metabolites in individuals with constipation-predominant irritable bowel syndrome
Source: Front Microbiol. 2025 Sep 4;16:1617288. doi: 10.3389/fmicb.2025.1617288 (PMC12445051; doi:10.3389/fmicb.2025.1617288)
Supplement: Supplementary file 1 [file Table_1.docx]

Supplementary Table 1. The detailed information of the materials

| Reagent | CAS | Purity | Brand |
| --- | --- | --- | --- |
| H_2_O | 7732-18-5 | LC-MS | Thermo |
| Acetonitrile | 75-05-8 | LC-MS | Thermo |
| Methyl alcohol | 67-56-1 | LC-MS | Thermo |
| Isopropanol | 67-63-0 | LC-MS | Thermo |
| Ammonium acetate | 73594 | LC-MS | SIGMA |

| Instrument | Type | Brand |
| --- | --- | --- |
| High performance liquid | ExionLC | AB Sciex |
| High-resolution mass spectrometry | TripleTOF 6600+ | AB Sciex |
| Chromatographic column | Waters HSS T3 column | Waters |
| Centrifugal machine | Legend Micro 17R | Thermo |
