# Supplementary figures and images for: Characterization of gut microbiota and metabolites in individuals with constipation-predominant irritable bowel syndrome
Source: Front Microbiol. 2025 Sep 4;16:1617288. doi: 10.3389/fmicb.2025.1617288 (PMC12445051; doi:10.3389/fmicb.2025.1617288)

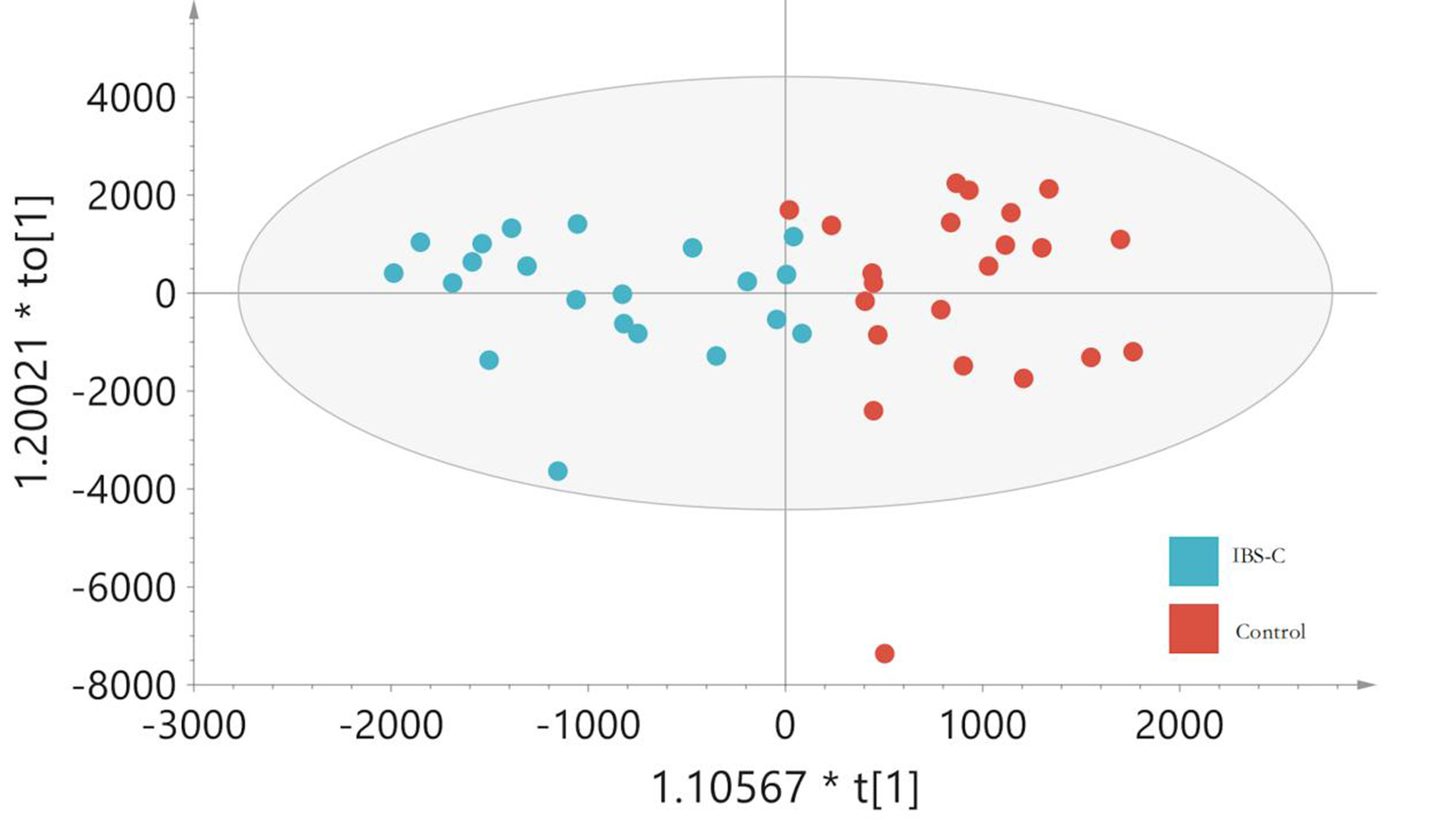

Supplement: Supplementary file 2 [file Image_1.jpeg]

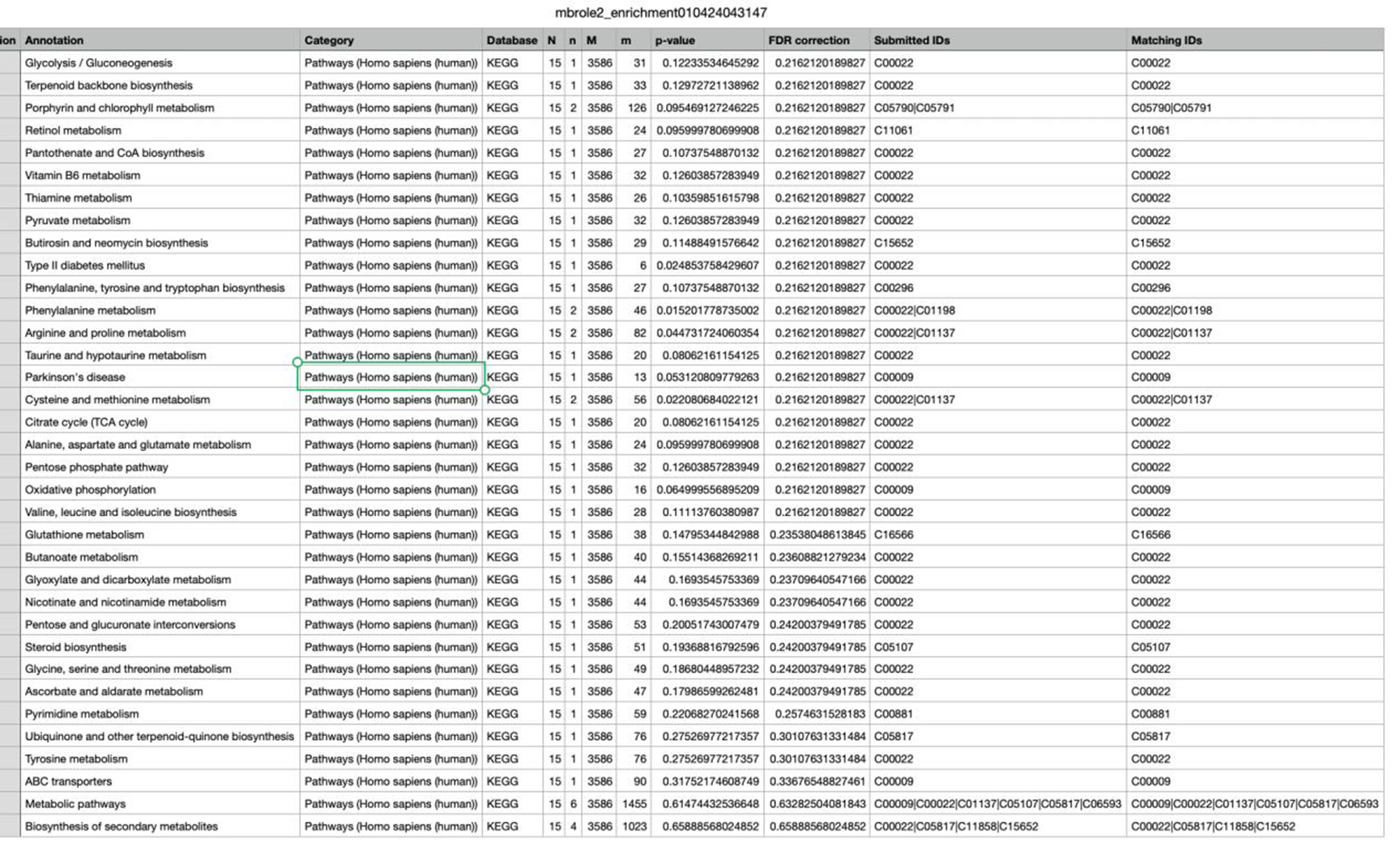

Supplement: Supplementary file 3 [file Image_2.jpeg]
